# Effect of spinal orthosis on clinical outcomes of patients after oblique lumbar interbody fusion: a randomized controlled trial study protocol

**DOI:** 10.1186/s13063-023-07796-x

**Published:** 2023-12-04

**Authors:** Nian-rong Han, Akram Osman, Wei Hu, Yi-fei Huang, Yan-lu Liu, Zhan-jun Ma

**Affiliations:** Xinjiang Medical University, 393 Xinyi Rd, Xinshi District, Ürümqi, Ürümqi, 830011 Xinjiang China

**Keywords:** Oblique lumbar interbody fusion, OLIF, Spinal orthosis, Lumbar minimally invasive surgery, Clinical efficacy

## Abstract

**Background:**

Oblique lumbar interbody fusion (OLIF) is an internationally popular minimally invasive technology for the treatment of various lumbar diseases. Since its introduction to China in 2014, OLIF technology has clearly shown its superiority in reconstructing intervertebral stability, restoring intervertebral space height, achieving indirect decompression, and restoring normal lumbar sequence. However, some patients still suffer from persistent symptoms after OLIF, including low back pain and soreness, which indirectly affect the overall surgical efficacy and patient satisfaction. Therefore, some clinicians recommend that patients routinely use spinal orthoses after OLIF to reduce the stress on the lower back muscles and ligaments, thereby relieving or avoiding postoperative residual symptoms or new symptoms. Accordingly, spinal orthosis use after OLIF has emerged as an essential option. However, the role of spinal orthoses in OLIF and their specific impact on postoperative patient clinical outcomes have remained unclear, and there is a lack of strong clinical evidence to indirectly or directly support the role of spinal orthoses in OLIF and demonstrate their impact on patient clinical outcomes. This study aims to investigate the role of spinal orthoses in OLIF by grouping patients based on the use or nonuse of spinal orthosis after OLIF, thus providing a better basis for the majority of patients and physicians.

**Methods/design:**

We plan to conduct a 1-year randomized controlled trial involving 60 subjects. The subjects will be randomized into two groups: group A (those wearing spinal orthoses after surgery) and group B (those not wearing spinal orthoses after surgery). The clinical outcomes of these patients will be evaluated using the Oswestry disability index, visual analog scale, and Brantigan, Steffee, Fraser 1 day before surgery and 2 weeks and 1, 6, and 12 months after surgery.

**Discussion:**

This randomized controlled trial aims to provide a reference for further comprehensive trial design. The findings of this study will provide a better and more scientific basis for the choice of postoperative rehabilitation and treatment for patients undergoing such a procedure.

**Trial registration:**

This study has been registered in the Chinese Clinical Trial Registry (Registration No.: ChiCTR2200059000). Registration date: April 22, 2022.

Registration website: http://www.chictr.org.cn/showproj.aspx?proj=166310

**Supplementary Information:**

The online version contains supplementary material available at 10.1186/s13063-023-07796-x.

## Background

Oblique lumbar interbody fusion (OLIF) is an innovative minimally invasive technology to treat various lumbar spine diseases, which was first proposed by Mayer in 1997 [1]. It has not been widely applied due to the limitation of supporting devices and cages, until 2012, when Prof. Hynes modified and developed a special fusion cage and its supporting access system based on the original transforaminal lumbar approach interbody fusion cage. OLIF technology does not destroy the posterior muscles, ligaments, or other structures, thus reducing the risk of postoperative low back pain, and also enables direct removal of a large number of diseased intervertebral disc tissues, thus allowing a cage with a larger contact area, which can greatly increase the support strength provided by the cage [2] and increase the success rate of fusion. Therefore, OLIF technology is recommended for various lumbar diseases that require intervertebral stability reconstruction, restoration of interbody height, interbody decompression, and restoration of normal lumbar sequences.

According to the recommendations of the Clinical Application Guidelines for Lumbar Oblique Lateral Interbody Fusion [3] of the Spine Surgery Group of the Chinese Orthopedic Association, the OLIF technology is intended for lumbar spinal stenosis and lumbar degenerative scoliosis based on imaging, correction of lumbar anterior lordosis in combination with posterior internal fixation, segmental instability and I degree lumbar spondylolisthesis, II degree lumbar spondylolisthesis, adjacent vertebral disease after lumbar fusion, and discogenic low back pain. However, it is not recommended for spinal stenosis caused by nucleus pulposus prolapse, fat deposition or other space-occupying factors, congenital spinal stenosis, bony spinal stenosis caused by calcification of the ligamentum flavum, or spinal stenosis caused by bony fusion of the posterior facet joints [4]. From the above indications and contraindications, it can be noted that OLIF technology is a classic and effective approach to treat spinal instability diseases [5–9]. OLIF technology has been compared with classical surgical methods such as transforaminal lumbar interbody fusion, anterior lumbar interbody fusion, and posterior lumbar interbody fusion in previous studies, and it was found that OLIF technology was superior in shortening the hospitalization time, reducing blood loss and postoperative low back pain, and restoring intervertebral space height and segmental lordosis [10–13]. With the continuous development of OLIF technology, the in-depth study of anatomy [14, 15], and based on a summary of clinical experience, the spine surgery team of the Affiliated Hospital of Zhejiang University in China modified the original OLIF surgical approach and proposed the anterior-inferior psoas (AIP) exposure technology [16, 17]. AIP technology, as a localized improvement of OLIF technology, reduces the risk of OLIF and makes the OLIF technology simple and clear, and is more suitable for Chinese patients, thus further promoting the wide clinical application of OLIF technology.

It is well established that any type of surgery is a form of trauma for human beings, regardless of the size of the trauma, but it is also likely to cause damage, and some such damage is even harmful. Despite many advantages of OLIF in lumbar spine surgery, it is also an invasive operation for the body, which may lead to residual symptoms or new complications in some patients after surgery and may indirectly increase the rate of lumbar reoperation and reduce the effect of surgery [18]. Therefore, it is imperative to adopt other means after lumbar surgery to reduce residual symptoms and postoperative complications. The commonly used clinical approaches include prolonged bed rest after surgery, avoidance of labor, lifting heavy articles, and prolonged standing or sitting and wearing a spinal orthosis. A better and commonly used clinical method to avoid reducing the quality of life of patients after surgery is to wear a spinal orthosis routinely after surgery.

Generally, fixation of any musculoskeletal injury is favorable for reducing the pain after injury [19]. Therefore, spinal orthoses can be used not only to relieve residual back pain symptoms [20] but also for postoperative rehabilitation of patients after lumbar spine surgery [21]. The use of spinal orthosis after OLIF is to fix the entire body movement [22] and the motion segment, relieve muscle strength [23], reduce pain [24], enhance the fusion rate [25], and improve the functional prognosis [26]. However, it is undeniable that non-standard or the wearing of spinal orthoses not for the prescribed time after surgery may also lead to muscle atrophy [27], skin irritation, delayed recovery, and other side effects.

For this reason, although the spinal orthosis exhibits some advantages after lumbar fusion, the necessity of its clinical use has been questioned due to its limitations. Additionally, the role of postoperative orthoses in OLIF has not been determined, and there is a lack of strong evidence to prove any correlation between the routine use of orthoses after OLIF and the final clinical outcome of patients. Therefore, we plan to conduct a randomized trial to evaluate the necessity of using lumbar orthoses after OLIF for postoperative adjuvant therapy in such patients.

### Assumption

It is assumed that the clinical outcome of patients wearing spinal orthoses after OLIF is superior to that of patients in the control group.

## Methods/design

This is a randomized controlled trial, with the aim of comparing the effects of spinal orthosis on the clinical outcomes of patients after OLIF. This study was approved by the Ethics Committee of the Fourth Clinical Medical School of Xinjiang Medical University and will be conducted in accordance with the *World Medical Association Declaration of Helsinki*. This study has been registered in the Chinese Clinical Trial Registry (Registration No.: ChiCTR2200059000). Before treatment, all patients should sign the informed consent form.

**Fig. 1:**
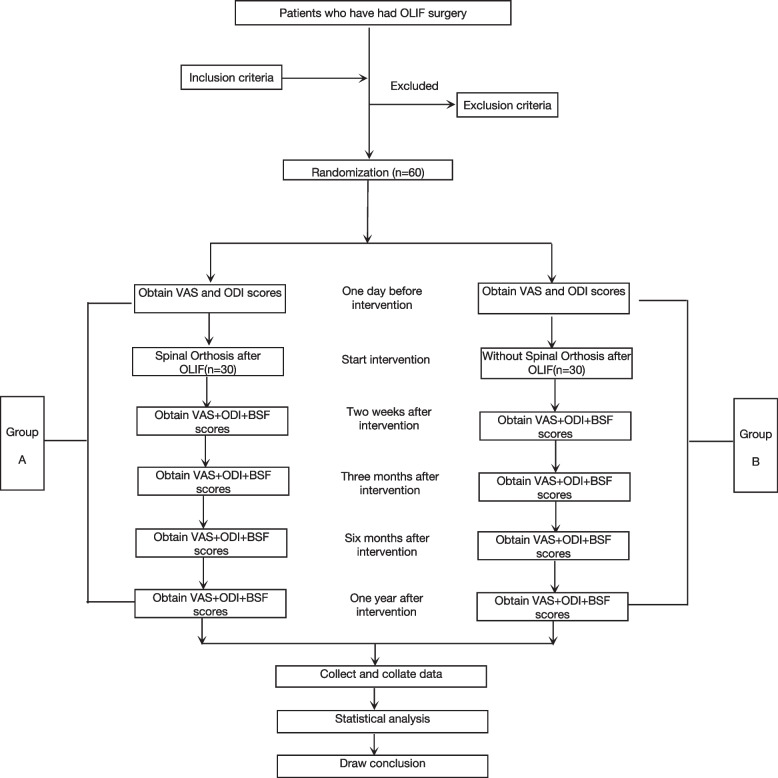
Study flow chart. OLIF, oblique lumbar interbody fusion; VAS, visual analog scale; ODI, Oswestry disability index; BFS, Brantigan, Steffee, Fraser

The evaluation and statistical analysis of the results of this study will be performed by professionals who are not involved in the grouping. The study flow is shown in Fig. 1. The patients will be randomized into two groups, that is, group A (those wearing spinal orthoses after surgery) and group B (those not wearing spinal orthoses after surgery; control group).

The main outcome measures include the Oswestry disability index (ODI), visual analog scale (VAS), and Brantigan, Steffee, Fraser (BSF) to assess pain intensity [28]. The clinical outcomes of these patients will be assessed using ODI and VAS scores 1 day before surgery and 2 weeks and 3, 6, and 12 months after surgery, and BFS scores will also be assessed starting from 2 weeks after surgery.

### Recruitment

First, we will evaluate potential candidates and inform them of our study procedures. Eligible patients will be randomized into two groups after written informed consent is obtained from each patient. Treatment will be given accordingly to the patient group. Patient recruitment and grouping will be performed by physicians in the subject group.

### Sample size

Because this study is based on clinical application, it is difficult to calculate the sample size through pre-experiments according to the actual situation; thus, the sample size of this study was finally determined based on the previous relevant literature data. The sample size outcome effect size was obtained from the literature using the ODI and VAS scores [29]. The mean change of the ODI in group A was approximately 92%, and the mean change of the VAS was approximately 0–3 points, while the mean change of the ODI in group B was approximately 85%, and the mean change of the NRS was approximately 4–5 points. The sample size was calculated using the sample size formula of the quantitative equivalence experiment *n* = 2(*Z*_*α*_ + *Z*_*β*_)^2^*δ*^2^/(*D* − *Δ*)^2^, assuming that *α* = 0.05 and *D* = 2.09 − 1.45 = 0.64, *δ*^2^ = [(0.56 + 0.78)/2]^2^ = 0.46 was obtained from literature retrieval, different values of *β* and superiority bounds were selected to calculate the sample size matrix, as shown in Table 1, and finally *n* = 50 was selected; taking into consideration a 20% dropout, thus 30 cases should be enrolled to each group, a total of 60 cases in two groups.

**Table 1:** Sample size matrix

*α*	*β*	*D*	*δ*^*2*^	*Δ*	*n*
0.05	0.2	0.64	0.46	0.2	38
0.05	0.2	0.64	0.46	0.3	63
0.05	0.1	0.64	0.46	0.2	50
0.05	0.1	0.64	0.46	0.3	84

This trial is to be conducted in the Second Spine Department of the Fourth Clinical Medical School of Xinjiang Medical University, and hospitalized patients who meet the inclusion criteria will be included in the study. Because of the large number of patients undergoing OLIF in this department, it is possible to recruit sufficient subjects within the prescribed time; thus, an insufficient sample size is highly unlikely. In addition, there are benefits for patients participating in the study to supplement the sample size (e.g., free professional consultation, postoperative functional exercise instruction, and postoperative care consultation).

### Inclusion, exclusion, and diagnostic criteria

The inclusion criteria are shown in Table 2, and exclusion criteria are presented in Table 3. All included patients who are indicated for surgery will be screened in strict accordance with the inclusion and exclusion criteria. The diagnosis of related diseases should be determined by physicians with extensive clinical experience based on the corresponding imaging examination, physical examination, and recognized standards and should be strictly controlled for accuracy in meeting the inclusion criteria and not meeting the exclusion criteria.

**Table 2:** Inclusion criteria

1) Patients with grade I or II spondylolisthesis according to the examination
2) Those diagnosed as discogenic low back pain, disc herniation, or lumbar spinal stenosis by imaging examination
3) Those with pain caused by the disease that seriously affects daily work and life and is not relieved after a short period of conservative treatment
4) Those with a lesion responsible segment ranging from the second lumbar vertebra (L2) to the first sacral vertebra (S1) and a single responsible space
5) Those aged between 18 and 80 years
6) Those with a disease duration ≥ 6 months

**Table 3:** Exclusion criteria

1) Patients with spinal fractures, spinal infections, and intraspinal tumors
2) Those who previously underwent spinal surgery
3) Those with osteoporosis (T < − 2.5), systemic autoimmune diseases, end-stage renal disease, and Parkinson’s disease
4) Those with combined major medical diseases not suitable for surgery
5) Those who suffer from mental illnesses that prevent them from communicating properly or from strictly following medical advice

### Randomization and blinding

Physicians Zhan-jun Ma and Yan-lu Liu, from the Fourth Affiliated Hospital of Xinjiang Medical University, will use computer software to generate random sequences, which will be sealed in envelopes. Eligible patients will be recruited by physicians of the department, Akram Osman and Nianrong Han. The recruited patients will be randomized into two groups by Wei Hu, a physician of the department. In order to avoid the predictability of random sequences, the envelopes used are all of the same kind of envelope produced by the same manufacturer (that is, the appearance, thickness, texture are the same), and all sequences are not disclosed to recruiters and allocations. In this study, a single-blind approach will be adopted, that is, participants do not know which treatment they are receiving. The specific procedures performed will be kept confidential to all participants until unblinded.

To ensure the scientific accuracy of the study, it is also planned to charge the same fee for all subjects. The specific charges are as follows: preoperative preparation fee, surgery fee, anesthesia fee, cost of related equipment used during operation, and cost of intraoperative and postoperative drugs. In particular, the spinal orthoses used by the patients in the experimental group will be uniformly distributed by the research team to ensure that the spinal orthoses used by the patients in the experimental group are the same; thus, the cost of spinal orthoses will not be included. The total cost is RMB 55969.32.

### Intervention measures

The patients included in the research group will be randomized into two groups: group A (those undergoing OLIF + wearing spinal orthoses 3 days after surgery) and group B (those not suitable for spinal orthoses after surgery). VAS and ODI scores will be assessed and recorded for all patients 1 day before surgery. According to the inclusion criteria, all patients who undergo this procedure will have a single responsibility space and posterior four-screw implantation.

### OLIF surgical technique

Under general anesthesia, the patient is placed in the right lateral position and the surgical vertebral body is determined under X-ray fluoroscopy. With the responsible surgical space as the center, a surgical incision of approximately 6 cm is made along the body surface projection of the anterior edge of the upper vertebral body, the skin and superficial fascia are incised to bluntly separate external oblique, internal oblique, and transversus abdominis muscles, and the peritoneum is separated forward, which can reach the psoas muscle. The anterior space of the psoas major is bluntly separated with the fingers, and the psoas major is pushed dorsally to expose the surgical vertebrae. The automatic distractor is used for distraction, the camera source is installed, and the surgical intervertebral space is determined by fluoroscopy again. The annulus fibrosus is cut crossly with a sharp knife. The nucleus pulposus tissue and annulus fibrosus of the surgical intervertebral disc are removed using nucleus pulposus forceps. The intervertebral space is treated with different specifications of reamer in turn to break through the contralateral annulus fibrosus, and the intervertebral space is completely cleared to the bilateral cartilage endplate. The appropriate size of the test mold is selected. The intervertebral space is cleaned with saline and an interbody cage rich in allograft bone is implanted into the intervertebral space. A large amount of normal saline is used for flushing until there is no active bleeding, and gelatin sponge blood is filled. The fascia and subcutaneous tissue of the external oblique muscle is sutured using absorbable sutures layer by layer, and the wound is bound using medical adhesives.

The patient’s position is changed to the prone position, and after the bilateral pedicle sites of the operated vertebrae are determined by the “G” arm, a 1.5-cm incision is made on the both sides and an incision of approximately 1.5 cm in length is transversely made, respectively. Under the “G” arm fluoroscopy, the bilateral pedicles of the surgical vertebrae again are determined based on ortho-lateral images, the needles are inserted along the bilateral pedicles, and the guide wire is inserted. Minimally invasive hollow polyaxial screws are placed under the guidance of guide wires, and the rods are placed on both sides. The screws are screwed into the screw plugs and subsequently alternately lifted and replaced and locked with pressure. A large amount of normal saline is used for flushing until there is no active bleeding. The skin is sutured intradermally using absorbable sutures layer by layer, and the wound is bound using medical adhesives.

**Fig. 2:**
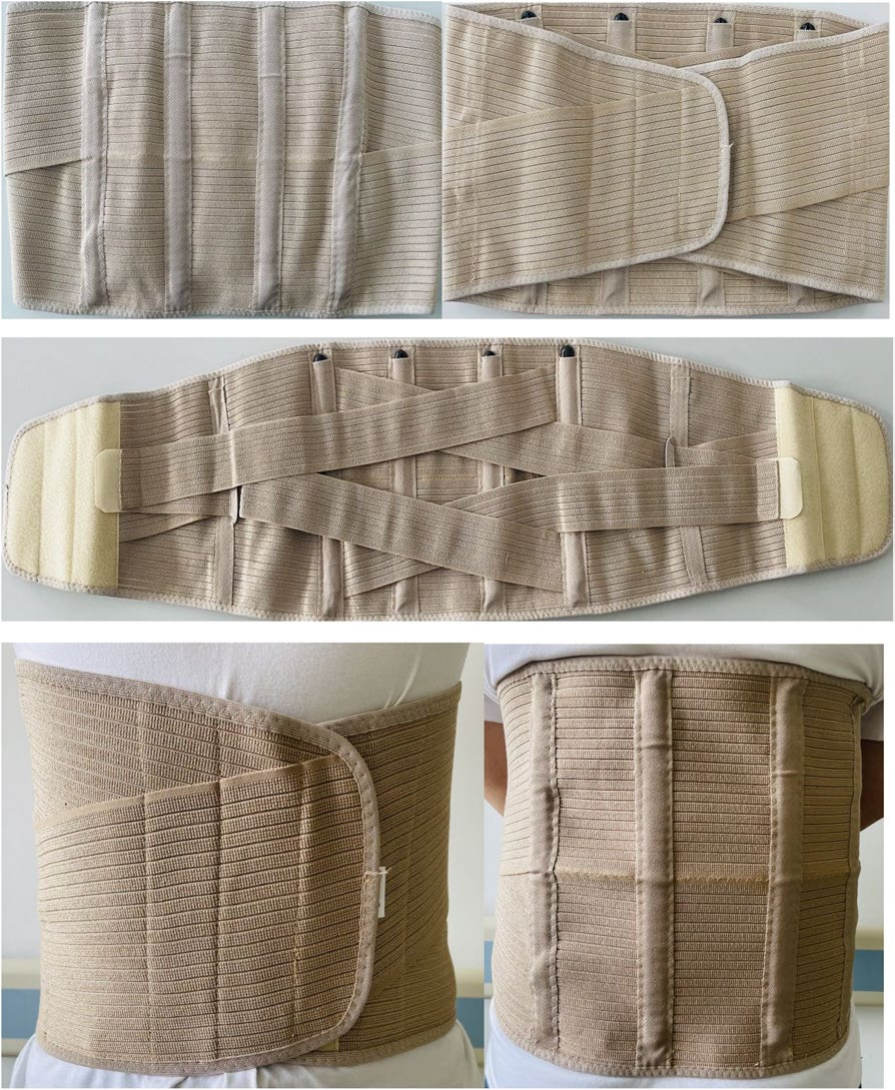
Spinal orthoses and manner of wearing

It should be ensured that both groups undergo the standardized OLIF procedure described above, and spinal orthoses are used in group A (experimental group) patients from 3 months after surgery except when bathing and lying in bed. The spinal orthosis used in the experimental group and the way of wearing it are shown in Fig. 2. The spinal orthoses are uniformly distributed by the research group, and patients are also instructed to wear them according to the unified standard.

### Data collection

VAS and ODI scores are assessed 1 day before surgery and 2 weeks, 3 and 6 months, and 1 year after surgery, and BFS scores will be assessed at 1, 3, and 6 months and 1 year after surgery to comprehensively evaluate the therapeutic effect. The treatment regimen and effect evaluation are illustrated in Table 4.

**Table 4:** Treatment regimen and effect evaluation

Data collection/recording	Person in charge	Observational phase	Interventions	Follow-up phase			
		1 day before surgery	Surgery	2 weeks after surgery	3 months after surgery	6 months after surgery	1 year after surgery
Obtaining informed consent	Yan-lu Liu	×					
Collecting patients’ basic information and medical history	Akram Osman Nianrong Han	×					
Screen patients for eligibility	Wei Hu Akram Osman	×					
Interventions	Yi-fei Huang		×				
ODI	Yan-lu Liu Zhan-jun Ma	×		×	×	×	×
VAS for pain	Yan-lu Liu Zhan-jun Ma	×		×	×	×	×
BSF score	Yan-lu Liu Zhan-jun Ma			×	×	×	×

*ODI*, Oswestry disability index; *VAS*, visual analog scale; *BFS*, Brantigan, Steffee, Fraser

### Main result measurement

A variety of outcome measures are used in this study, including the VAS (0–10 points) and ODI (0–50%), and are evaluated 1 day before surgery and 1, 3, and 6 months and 1 year after surgery. The ODI is used to assess disability associated with low back pain. The ODI has 10 questions about daily activities, including pain intensity, personal care, weight lifting, walking, sitting, standing, sleeping, sex, social life, and travel, each of which is rated on a scale of 0 to 5 points. The lower the score, the less affected the person is by pain. The VAS uses 0 points to indicate no pain and 10 points to indicate the peak of pain within the patient’s perception range, and the VAS is a self-assessment tool for pain consisting of 0 and 10 at either end of a straight line. Participants mark a point on the line to indicate their average pain level. The above two scales have been recognized by the medical community, and the value and effectiveness of the VAS and ODI scales have been demonstrated by related studies [30, 31]. The BSF score ranges from 0 to 4, where 0 points indicate no connection between the upper and lower parts of the vertebral body, height loss, and bone grafting absorption and 4 points indicate complete fusion and good shaping. The higher the score, the greater the degree of fusion. The main outcome measures in this study are the ODI, VAS, and BFS scores after follow-up and efficacy evaluation obtained as required. The above scores will be obtained by the designated personnel through follow-up visit. After obtaining the scores of the two, the efficacy is evaluated according to Table 4. Because the final efficacy evaluation of each patient is significantly affected by the above scores, which indirectly plays a decisive role in the results of the study, the relevant data should be obtained in strict accordance with the requirements during the follow-up process. The specific efficacy evaluation criteria are stated in Table 5.

**Table 5:** Efficacy evaluation

	Efficacy			
	Cured	Significantly effective	Taking effect	Ineffective
Evaluation index				
ODI score	≤ 40%	40–59%	60–70%	> 70%
VAS score	< 2	2–3	4–5	> 5
BSF score	≥ 3	2	1	0

*VAS*, visual analog scale; *ODI*, Oswestry disability index; *BFS*, Brantigan, Steffee, Fraser

### Monitoring, safety, and quality control

During the follow-up period, paper case report forms will be used to collect data. All data collection will be monitored in strict accordance with the standard procedures of the clinical trial center to ensure full compliance with International Coordinating Council standards and Good Clinical Practice principles. Any adverse events that occur in patients during the study period, such as worsening of clinical symptoms or loss of daily activity, will be fully evaluated through a doctor’s inquiry, physical examination, and imaging studies, and if adverse events are considered to be caused by intervention measures, the study will be discontinued and the patient will be treated symptomatically. Relevant data about the adverse events and the intervention measures in this study will be analyzed to improve the research measures. All adverse events will also be reported to the investigators. Due to short study period and low risk, we will not set up a trial steering committee. Any unexpected event will be reported directly to the investigator for comment. There is no interim analysis in this protocol, and Dr. Wei Hu has the right to make the final decision on whether to terminate the trial.

Other treatment methods allowed in the control and experimental groups are as follows. (1) Both groups of patients can receive routine nursing treatment in the Second Spine Department of Xinjiang Uygur Autonomous Region Hospital of Traditional Chinese Medicine, including daily inquiries about the patient’s condition, pain improvement, and patient care. All nursing programs will be performed by the designated nurses of the Second Spine Department. (2) The acupuncture treatment for patients in the two groups after OLIF will be performed by the designated physician of the acupuncture department of the hospital. Additionally, the lumbar standardized acupuncture treatment is adopted, that is, all subjects have the same acupuncture treatment. (3) Traditional Chinese medicine treatment is also allowed as adjuvant therapy, and the patients in both groups receive the same treatment. Therefore, an effect of the above treatment on the overall trial results can be avoided. Other treatment methods prohibited for the control and experimental groups are as follows. (1) It is prohibited to perform other invasive treatments on the lumbar spine. (2) Subjects are prohibited from taking medications that can improve the related clinical symptoms. (3) No additional treatment may be given to patients included in the study without the consent of the investigator.

Yan-lu Liu, a physician of Second Spine Department, had an informed conversation with each subject 2 days before surgery, and all subjects signed the informed consent form. After Dr. Yan-lu Liu obtained the informed consent of each subject, Dr. Nianrong Han from the Second Spine Department obtained from each subject the basic information and relevant medical history required for the trial, which was completed 2 days before surgery. Zhan-jun Ma, a physician of the Second Spine Department, checked whether the subjects met the prescribed inclusion criteria on the day before surgery and finally determined whether they were included in the trial. After Dr. Yan-lu Liu and Dr. Nianrong Han completed their work, investigator Akram Osman assessed the VAS and ODI scores 1 day before surgery. The OLIF surgery in this study was performed by Professor Yifei Huang, the chief physician of the Second Spine Department, the Fourth Clinical Medical College of Xinjiang Medical University, according to the requirements of this study. Later, the follow-up data of the ODI, VAS, and BFS scores were collected by Dr. Zhan-jun Ma and Dr. Yan-lu Liu. Importantly, for the same subject, the data should always be tracked and collected by the same person and cannot be performed alternately. Follow-up data collection should be completed within 24 h of the specified date. Dr. Akram Osman is responsible for sorting and entering the collected data into the specified data acquisition form and setting the password for independent storage. This work is completed within 24 h of receiving the data.

### Dropout or loss to follow-up

All patients have the right to withdraw from the study at any time. If a patient chooses to withdraw from the study, we will revoke the patient’s consent and terminate the study participation. The chief investigator may also terminate a subject’s participation if the subject violates the rules of management for this study or has a serious adverse event. Data of these subjects will be excluded in the final analysis.

### Statistical analysis

The Epidtat3.0 software is adopted to enter the observation data by two members of the research group alone, and SPSS18.0 is applied for statistical analysis. Demographic and clinical characteristics (such as gender, age, and weight) of all subjects will be processed based on descriptive analysis. Quantitative data will be expressed as the mean, standard deviation, median, and range and qualitative data will be presented as frequencies and percentages. Chi-square test will be used to compare the incidence of related adverse events between the two groups.

### Publication of experimental results

The study results will be published in Trials. The chief investigator and members of the study management team and other researchers will prepare the manuscript and submit it to the journal for publication. The final result of the manuscript is available to all after publication in the journal. If there are colleagues who need raw data or patient disclosure data, they can contact the corresponding author to request it.

## Discussion

According to previous studies and clinical experience, spinal orthosis is commonly used after open lumbar fusion. However, as previously reported, there is insufficient evidence to systematically prove the necessity of using spinal orthoses after lumbar spine surgery [32]. Obviously, there is a clear lack of consensus on the indications for routine spinal orthosis use after lumbar spine surgery.

For lumbar fusion, the benefits of postoperative bracing are intensively debated. In clinical practice, due to the absence of scientific guidelines, some physicians often subjectively choose whether to use a spinal orthosis after OLIF surgery based on their own experience. This subjectivity may increase the possibility of some unnecessary risks, indirectly leading to undesirable outcomes and increasing uncertainty about prognosis, and may also increase the pain and economic burden of patients.

Patients who use spinal orthoses after surgery may feel more comfortable and, thus, move more easily under ordinary circumstances. However, there are no definitive findings on whether routine spinal orthosis use after surgery leads to any negative effects and thus adversely affects the final outcome. Accordingly, we have designed this study to prove whether routine spinal orthosis use in patients after OLIF has an impact on their clinical outcomes, so as to provide a more scientific basis for clinicians on deciding on its use.

Clearly, this study has some limitations. The clinical outcome evaluation indexes are relatively single, and the follow-up time is relatively long, which may lead to problems such as a reduced reliability of some data. Therefore, the data are collected in strict accordance with the requirements during the study, and the relevant factors affecting the experimental data are minimized and the existing limitations will be further improved in the future research process.

To our knowledge, this study is the first prospective randomized trial to evaluate the effect of spinal orthosis on postoperative clinical outcomes in patients undergoing OLIF, thus providing clinicians and patients with a more scientific treatment choice.

### Trial status

The version number of this protocol is as follows: no. 3.0, date: May 8, 2022. The date of subject recruitment is as follows: April 1, 2023, and it is expected to complete all research processes by the end of April 2024.

### Supplementary Information

**Additional file 1.**

## Data Availability

Only the authors AO and HW will have access to the final trial dataset, while the rest are prohibited from accessing the data. Any data required to support the protocol can be supplied on request. The datasets analyzed during the current study and statistical code are available from the corresponding author on reasonable request, as is the full protocol.

## Abbreviations

OLIF: Oblique lumbar interbody fusion
VAS: Visual analog scale
BFS: Brantigan stefee fraser
TLIF: Transforaminal lumbar interbody fusion
ALIF: Anterior lumbar interbody fusion
PLIF: Posterior lumbar interbody fusion
AIP: Anteroinferior psoas
